# Editorial: Multi-Omics Technologies for Optimizing Synthetic Biomanufacturing

**DOI:** 10.3389/fbioe.2021.818010

**Published:** 2021-12-15

**Authors:** Young-Mo Kim, Christopher J. Petzold, Eduard J. Kerkhoven, Scott E. Baker

**Affiliations:** ^1^ Integrative Omics Group, Biological Sciences Division, Pacific Northwest National Laboratory, Richland, WA, United States; ^2^ Department of Energy, Agile BioFoundry, Emeryville, CA, United States; ^3^ Biological Systems and Engineering Division, Lawrence Berkeley National Laboratory, Berkeley, CA, United States; ^4^ Department of Biology and Biological Engineering, Chalmers University of Technology, Gothenburg, Sweden; ^5^ Functional and Systems Biology Group, Environmental Molecular Sciences Division, Pacific Northwest National Laboratory, Richland, WA, United States

**Keywords:** biomanufacturing, multi-omics analysis, synthetic biology, DBTL cycle, metabolic engineering

Industrial manufacturing endures as an essential human activity yielding a variety of useful products; it plays a significant role in the global economy with huge impacts in everyday life. However, the manufacturing process requires consumption of various raw materials (especially petroleum derivatives), generates a variety of harmful waste products, causes pollution, and is energetically inefficient. Biological manufacturing from sustainable, affordable, and scalable feedstocks potentially enables the displacement of the entire portfolio of currently available products produced by industrial processes, enabling the manufacturing of renewable and eco-friendly products (Clomburg et al., 2017). Thus, successful development of a robust biomanufacturing strategy and technology platform, based on the latest advances in synthetic biology and chemical catalysis, will decrease both the cost and production time compared with previous manufacturing processes. Development of biomanufacturing processes using a synthetic biology platform requires the multidisciplinary efforts of science and engineering fields including molecular biology, microbiology, genetic engineering, informatics, metabolic modeling and chemical or process engineering (El Karoui et al., 2019).

**FIGURE 1:**
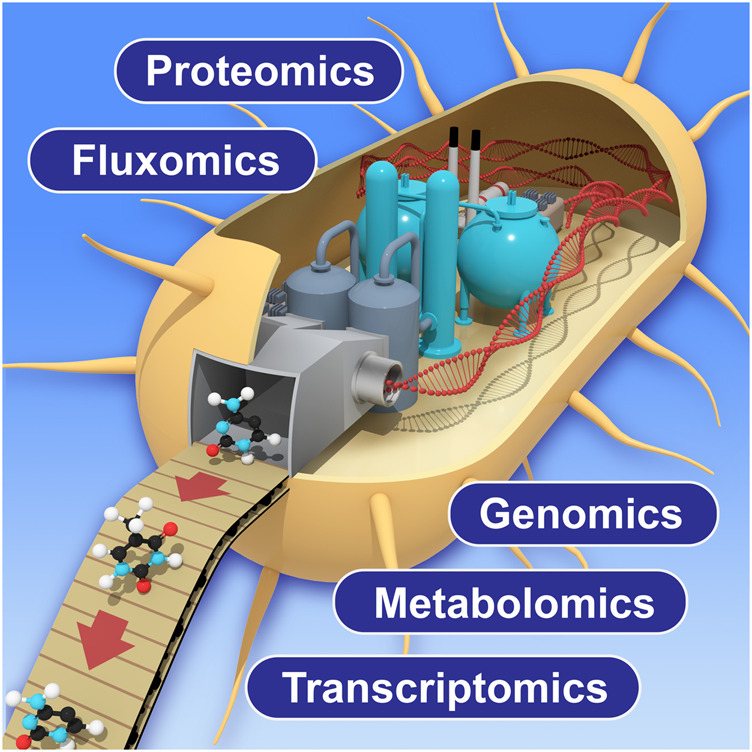
Schematic view of multi-omics application to biomanufacturing process. Improvement of each technology will enhance the measurement coverage and accuracy in future applications.

In this research topic, Amer and Baidoo discussed the importance of using multi-omic analytical approaches to monitor and improve the biomanufacturing process. These approaches include genomics, transcriptomics, proteomics, metabolomics and fluxomics (Figure 1). The multi-omics data acquired from the biomanufacturing process not only provides potential solutions to low production efficiency by identifying underlying metabolic bottlenecks or pathway sinks, but also guides the understanding of how these modified biological systems function. Furthermore, such multi-omics technologies are constantly innovated and improved to expand molecular detection coverage, obtain data with increased accuracy by using new or novel analytical instruments, achieve better computational algorithms, and create wider and deeper databases to support a growing variety of biological host systems. Roy et al. described a combined computational tool to optimize the DBTL (Design-Build-Test-Learn) cycle in biomanufacturing process by collecting, visualizing, and utilizing large multi-omics datasets from various biological systems and emphasized their importance in the following metabolic engineering processes with machine learning approaches.

Gao et al. compared microflow and nanoflow liquid chromatography-selected reaction monitoring (LC-SRM) methods for analysis of hundreds of targeted peptides associated with 132 proteins in major pathways of *Pseudomonas putida,* a versatile bacterial host for production of bioproducts and biofuels *via* metabolic engineering. The increased throughput and accuracy of protein measurement will not only reduce the DBTL cycle time in future applications, but is, in addition, easily applied to other biomanufacturing host organisms.

Fletcher and Baetz reviewed the toxicity of phenolic compounds which are produced from pretreatment or hydrolysis of natural lignocellulosic biomass based on functional genomics and transcriptomics approaches, especially to the important model organism and industrial bioproduction host strain, *Saccharomyces cerevisiae*. Information regarding physiological tolerances of toxic phenolic compounds may be applied and evaluated in other host strains for future improvement. In that regard, Garcia et al. developed the genome-scale metabolic model of *Clostridium thermocellum* for efficient conversion of lignocellulosic biomass which has unique preference for its anaerobic and thermophilic growth attributes. This model will provide a useful tool to understand physiological and metabolic parameters associated with potential future biomanufacturing process.

Pinheiro et al. studied a xylose metabolism by *Rhodosporidium toruloides*, an oleaginous yeast with significant emerging potential in industrial applications, using a detailed physiological characterization interpreted with absolute proteomics and genome scale metabolic models. Kim et al. performed a multi-omics analysis on *R. toruloides* and the transcriptomics, proteomics, metabolomics and RB-TDNA sequencing data improved the current genome-scale model to make it a more exhaustive and accurate metabolic network model.

Pomraning et al. integrated high-throughput proteomics and metabolomics data as part of a DBTL cycle focused on improving production efficiency of 3-hydroxypropionic acid (3HP) in engineered *Aspergillus pseudoterreus* strains. This was the first report of 3HP production in a filamentous fungus amenable to industry-level biomanufacturing of organic acids at high titer and low pH. Chroumpi et al. studied another filamentous fungus *Aspergillus niger* for better understanding of pentose catabolic pathways by deletion of the key genes. The high-throughput multi-omics data (i.e., transcriptome, metabolome and proteome) generated on the mutant strains revealed that these genes are critical for metabolic pathways but not as critical for growth of *A. niger* on more complex biomass substrates, which raises fundamental questions on nutrient acquisition during growth on various carbon sources.

Wu et al. investigated the metabolic potential of *Zymomonas mobilis* for conversion of glucose and xylose to 2,3-butanediol. This study used calculated thermodynamic and kinetic parameters to generate insights of *Z. mobilis* metabolism. They also performed pathway and dynamic flux balance analysis to understand metabolic potential and production efficiency for future industrial applications. Nitta et al. acquired metabolomics and transcriptomics data on antibiotic producing strain, *Streptomyces coelicolor* to understand the functional connections between the production of antibiotic, actinorhodin and the level of cAMP. They found that higher levels of cAMP improved cell growth and production of actinorhodin, which was confirmed by the metabolomic and transcriptomic data.

We conclude by emphasizing that high-throughput multi-omics data play a critical role to unravel the complexities of metabolic engineering to improve production efficiency and product titer produced by a variety of industrial microbes. In addition, generation of multi-omics datasets accelerates the adoption and subsequent application of artificial intelligence approaches such as machine learning to design of improved microbial bioproduction host systems (Lawson et al., 2021). In terms of technological perspectives, enhanced high-throughput measurement and improved coverage of multi-omics analyses with higher accuracy will not only benefit in shortened DBTL cycle times for the metabolic engineering process, but also will lead to improved fundamental understanding of engineered biosystems. Refining tools and analytical platforms will benefit manipulating, modifying, and reshaping potential host systems. The long-term outcomes of these efforts will impact the world and our future by decarbonizing the current manufacturing processes *via* an environmental-friendly manner.
